# A randomized controlled trial to evaluate the effectiveness and safety of electro acupuncture and transcranial direct current stimulation with computerized cognitive rehabilitation in patients with vascular cognitive impairment

**DOI:** 10.1097/MD.0000000000021263

**Published:** 2020-07-17

**Authors:** Hyeng Kyu Park, Min Keun Song, Jae Hong Kim, Jae Young Han

**Affiliations:** a Department of Physical and Rehabilitation Medicine, Chonnam National University Medical School and Hospital, Gwangju City; b Department of Acupuncture and Moxibustion Medicine, College of Korean Medicine, Dongshin University, Naju City, Republic of Korea.

**Keywords:** computer-based cognitive rehabilitation, electroacupuncture, study protocol, transcranial direct current stimulation, vascular cognitive impairment

## Abstract

**Background:**

Vascular cognitive impairment (VCI) refers to all cognitive disorders caused by cerebrovascular disorders. For the treatment, many types of pharmacologic and nonpharmacologic treatments are used but their underlying mechanisms and effects are unclear. Regarding nonpharmacologic treatment, electroacupuncture (EA), transcranial direct current stimulation (tDCS), and computerized cognitive rehabilitation treatment (CCRT) are effective. Here, we report the protocol for a randomized controlled trial of the effect and safety of combination therapy of EA or tDCS and CCRT in patients with VCI.

**Methods:**

This study will be a prospective, outcome assessor-blinded, parallel-arm, randomized controlled clinical trial. Participants with cognitive impairment caused by stroke after 3 months of onset (n = 45) will be randomly assigned to a CCRT, combination therapy with EA and computerized cognitive rehabilitation treatment, or combination therapy with tDCS and computerized cognitive rehabilitation treatment group. All groups will receive treatment 3 times per week for 8 weeks, giving a total of 24 treatments. The CCRT group will perform a training task like shopping, calculating, and others and involving computerized cognitive assessment and brain training system (RehaCom) for 30 minutes. The combination therapy with EA and computerized cognitive rehabilitation treatment group will receive EA using 8 acupuncture points – baekhoe, sinjeong, both sides of pungji, 4 sites of sishencong – and will be applied using an EA stimulator and receive CCRT for 30 minutes at the same time. The combination therapy with tDCS and computerized cognitive rehabilitation treatment group will receive tDCS treatment and receive CCRT for 30 minutes at the same time. The primary outcome will be evaluated using the Lowenstein occupational therapy cognitive assessment, while other scales assessing walking ability, activities of daily living, and quality of life are considered secondary outcome measures. Outcomes will be evaluated before intervention, at the end of intervention 8 weeks after the first intervention, and 4 weeks after completion of the intervention program.

**Discussion:**

This study aims to examine the effect and safety of combination therapy with EA or tDCS and CCRT in patients with VCI. This study can be useful in developing new treatment technologies using collaborative research with combined traditional Korean and conventional medicines.

**Trial registration:**

This trial has been registered with cris.nih.go.kr (registration number, KCT 0003644 Registered 01 April 2019, http://cris.nih.go.kr).

## 1 Introduction

Vascular cognitive impairment (VCI) refers to cognitive dysfunction attributable to all forms of cerebrovascular disease.^[1]^ It ranges from mild cognitive impairment to dementia caused by cerebrovascular disease.^[2]^ Patients with VCI experience symptoms caused by prefrontal subcortical lesion; detrimental effects on attention, executive functions, planning, and cognitive control; and decreased verbal fluency and memory.^[3]^

A previous study reported that about half of patients with VCI (49%) progress to dementia and the mortality rate of patients with vascular dementia within 5 years as high as 60%.^[4]^ Though cerebrovascular causes of dementia account for approximately 20% to 30% of cases, the frequency of VCI may be higher than expected because of the low rate of treatment in patients with cerebrovascular disease.^[5]^

Alzheimer disease is the most prevalent form of dementia, followed by vascular dementia, which is becoming increasingly important because many cases of Alzheimer disease are associated with vascular factors.^[6]^ In particular, considering that the incidence of cerebrovascular disease and heart disease is increasing in the elderly population and considering the rapid societal aging in Korea, it will be a serious social burden on our society in the future.^[7]^

Since the risk factors for cerebrovascular disease are already known, and treatment and prevention is to some extent possible, treatment and prevention of vascular cognitive disorders resulting from cerebrovascular disease is relatively possible, in contrast to Alzheimer dementia or irreversible cognitive disorders.

Therefore, in patients with a vascular cognitive disorder who have mild cognitive impairment now, but have potential risk factors that can be aggravated, it may help with early diagnosis, prevention, and treatment.

Many clinical trials have reported that various medications are effective in improving vascular cognitive disorders, but there are few medications that are officially licensed.^[8]^ Although medical treatment has the advantage of improving some functional and behavioral aspects of a patient's life, the role of nonpharmacological treatment has primarily been seen as compensation for the limitations of pharmacological therapy because of the potential for controversy over side effects or uncertain efficacy.^[9]^

Electroacupuncture (EA) is a method of treatment that passes a weak current through an acupuncture stimulator after passing through 2 or more acupuncture points to deliver electrical stimuli with needle stimulation in oriental medicine and has been reported to improve cognitive impairment.^[10,11]^

Computerized cognitive rehabilitation is the treatment of a specific area that is affected in a patient using computer programs. It is used in the improvement of cognitive function because it is possible for the patient to self-practice, learn, and repeat the cognitive task. Moreover, the immediate and direct feedback on the result of the treatment is possible and the result of the training task can be saved and progress can be observed.^[12]^

In addition, among the methods that can affect brain activity, transcranial direct current stimulation (tDCS) is used in clinical medicine as a brain stimulation method that increases or decreases the activation of the brain cortex by applying a weak direct-current-generated stimulation between 2 electrodes located on the scalp.^[13]^ tDCS is easier to use than other brain stimulation methods, such as transcranial magnetic stimulation, because it applies stimulation to increase awareness of exercise training protocols or recovery in an environment for recovery and is widely used in special studies as part of the experimental design or evaluation.^[14]^

In this study, we aimed to develop therapeutic technology based on the combination therapy of EA or tDCS with computerized cognitive rehabilitation. Through this clinical trial, we aim to contribute to the diversity of treatment and prevention methods for dementia by developing efficient nonpharmacological treatment techniques through the use of EA, computerized cognitive rehabilitation, and tDCS for VCI.

## 2 Methods

### 2.1 Study design

This study is a randomized controlled clinical trial with a prospective, outcome assessor-blinded, parallel-arm, 1:1:1 allocation ratio, single-center design. This study is a pilot study comparing the effect between “EA with computerized cognitive rehabilitation” and “tDCS with computerized cognitive rehabilitation” to investigate the effect and safety of combination therapy of EA or tDCS with computerized cognitive rehabilitation. Participants who meet the inclusion and exclusion criteria will be randomly allocated into a computerized cognitive rehabilitation treatment (CCRT) group (n = 15), combination therapy with EA and computerized cognitive rehabilitation treatment (CECCRT) group (n = 15), and combination therapy with tDCS and computerized cognitive rehabilitation treatment (CTCCRT) group (n = 15), for a total of 45 participants. The CCRT group will receive computerized cognitive rehabilitation therapy, the CECCRT group will receive combined EA and computerized cognitive rehabilitation therapy, and the CTCCRT group will receive combined tDCS and computerized cognitive rehabilitation therapy. All groups will receive therapy once a day for 3 days a week (excluding Saturdays and Sundays) for 8 weeks, giving a total of 24 therapies scheduled during the hospitalization period at Chonnam National University Hospital.

The outcomes will be measured before intervention, at the end of intervention, 8 weeks after the first intervention, and 4 weeks after completion of the intervention. The change in cognitive function as assessed using the Lowenstein occupational therapy cognitive assessment (LOTCA) scale will be measured as the primary outcome. The changes in the performance of every day movements will be measured using the Korean version of the modified Barthel index (K-MBI) and changes in health-related quality of life will be measured using the European Quality of Life Five Dimension Scale (EQ-5D-3L). The K-MBI and EQ-5D-3L will be secondary outcomes in this study.

This clinical trial was conducted by the Korea Health Industry Development Institute in 2018, a research project on the development of therapeutic techniques for the prevention of dementia and combination therapy with computerized cognitive rehabilitation (HI18C0546).

**Figure 1:**
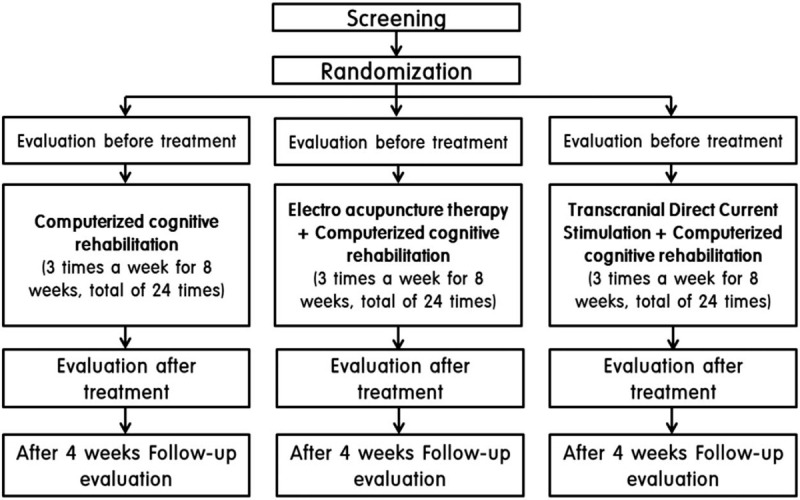
The flow diagram for this study.

The design of this study is summarized in Figure 1. And time table of protocol is summarized in Table 1.

**Table 1:**
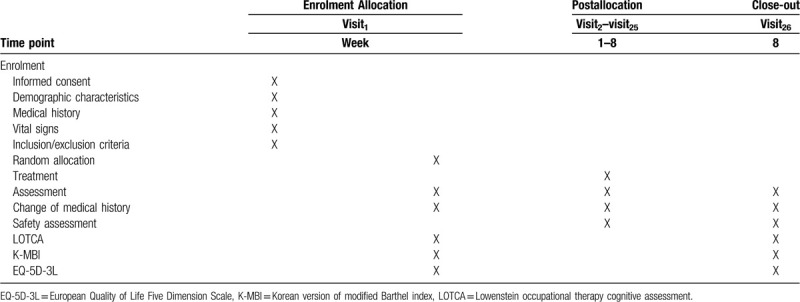
Time table of protocol.

### 2.2 Participant recruitment

Patients who have finished treatment of the early acute stage of stroke at the Department of Rehabilitation of Chonnam National University Hospital were enrolled. Patients confirmed as stable by a doctor and meeting the inclusion and exclusion criteria will be recruited.

All participants in this study will be given an explanation regarding this protocol by the clinical research coordinator (CRC) and will be able to voluntarily sign a consent form. The CRC will monitor the status of participants in this protocol to improve adherence.

### 2.3 Inclusion criteria

Patients will be considered for recruitment if they meet all of the following conditions: patients who are aged from 20 to 90 years, diagnosed with stroke by computerized tomography or magnetic resonance imaging examination, whose score on the Korean version of the Montreal Cognitive Assessment (MoCA-K) is below 22, and who can communicate with others and have no visual disturbance and no difficulty in reading and understanding Korean.

### 2.4 Exclusion criteria

Patients who cannot receive treatment due to general ill-health will be excluded. Additional exclusion criteria are as follows: cognitive impairment accompanied by brain tumor, encephalitis, or traumatic brain injury; significant cognitive impairment before stroke; severe depression or psychological disorders that make it difficult to adapt to treatment; change in the medication or other treatment that may affect the symptoms of cognitive impairment within the last month; contraindications to EA (eg, metal in the head or cochlea excluding that in the oral cavity [eg, electrode or stimulator, aneurysm clip or coil, stent, bullet piece, deep brain stimulator, vagus nerve stimulator, or ocular implant], artificial pacemakers, defibrillators, nerve stimulators, drug pumps, intracardiac intravenous line, continuous seizure, cranioplasty, ventriculoperitoneal shunt, sustained symptoms of headache, nausea, and vomiting associated with increased intracranial pressure); contraindications to scalp acupuncture (eg, wound or infections at the acupuncture site on the scalp, hemostatic abnormality due to abnormality of blood coagulation system such as hemophilia, and serious previous adverse reactions after acupuncture treatment); serious medical condition that leaves the patient unable to perform cognitive rehabilitation; musculoskeletal disorders or degenerative motor disorders such as Parkinson disease or Huntington disease that cannot complete a computer program; and pregnant women or women capable of childbearing.

### 2.5 Ethical considerations

This study will follow the guidelines of the Declaration of Helsinki for medical research involving humans.^[15]^ Ethical approval was obtained before participant recruitment and the Institutional Review Board of Chonnam National University Hospital has approved this study (CNUH-2019-061). All participants will receive sufficient information and explanation regarding the purpose and potential side effects of this study, and written informed consent will be asked from all participants before participating in this study.

### 2.6 Randomization

The treatment allocation into CCRT, CECCRT, or CTCCRT groups in this study will be determined using a randomization process. For this randomization, random allocation software^[16]^ will be used to randomly allocate 15 participants into each group. This randomization process will be conducted by a third-party team of statisticians and the allocations will be concealed and sequentially numbered with opaque and sealed envelopes. The serial number codes will be opened in the presence of the participants and CRC.

### 2.7 Implementation

Only the CRC will generate the allocation sequence, enrollment, and assign participants to interventions.

### 2.8 Blinding

This study is a prospective, single-blinded (outcome assessor), parallel-group, randomized controlled clinical trial with a 1:1:1 ratio of participants to be allocated into the CCRT or CECCRT or CTCCRT groups. In this study, each participant will know which program is applied to them. Therefore, we have adopted a single blinding (outcome assessor blinding) approach. To maintain the objectivity of this study, we have separated the outcome assessor, CRC, and therapist who treats the participant. In this study, individuals with conflicts of interest or preconceived positions are excluded to prevent related bias.

### 2.9 Intervention

All participants will receive CCRT in a sitting position, which focuses on cognition improvement. CCRT consists of screening and a treatment program, and these programs are available for pre and posttreatment evaluation. It is possible to automatically apply the treatment program according to the evaluation result and train a range of abilities from basic cognition (attention, memory) to higher cognition (metacognition). In addition, the real-time measurement automatically adjusts the difficulty according to the user's ability.

Participants will be selected for the program after screening tests have been conducted after enrollment. The screening test is composed of 5 types, and the training program is composed of 5 areas, giving in total 23 programs. Participants then perform the treatment tasks (shopping, scheduling, logical thinking, calculation) of the “executive functions” program of the computerized cognitive treatment system (RehaCom Training Test) for 30 minutes.

CECCRT group will receive EA treatment during the period of CCRT. EA will be performed in the sitting position at baekhoe (GV20), sinjeong (GV24), both sides of pungji (GB20), and 4 sites of sishencong (EX-HN1), in total 8 acupuncture sites according to a previous study.^[17]^ Acupuncture needles will be sterile, stainless steel, and disposable (size, 0.25 × 30 mm; Dong Bang Acupuncture, Inc, Boryeong, Republic of Korea; product no. A84010.02). During EA, guide tubes and an EA stimulator (CELLMAC PLUS [STN-330]; Stratek, Co, Ltd, Anyang, Republic of Korea; product no. A16010.04) will be used. The needles will be subgaleally inserted at an angle of 15° to 30° along the scalp at GV20, GV24, and EX-HN1. The needles will be directed in the direction of the tip of the nose at a depth of 17 to 40 mm at GB20. GV20 and GV24 will be inserted anteriorly direction along the scalp at a depth of 15 to 45 mm depth at GV20 and 15 to 24 mm at GV24. Anterior EX-HN1 will be punctured anteriorly, while the right, left, and posterior EX-HN1 will be punctured in the direction of GV20 at a depth of 9 mm.^[18]^ During CECCRT, manual stimulation will not be used. GV24 and GV20, left and right EX-HN1, anterior and posterior EX-HN1, and left and right GB20 will be subjected to EA under the following parameters: continuous waves; frequency, 3 to 15 Hz; and intensity, 2 to 4 mA for 30 minutes.^[17]^

The CTCCRT group will receive tDCS treatment during the CCRT period. The tDCS treatment will be conducted in the sitting position as follows. Two electrodes and a HDC stimulator (HDC kit, YoungWoo med, Korea) will be used to deliver 1 mA of tDCS to the skull. The anode is attached to F3, the anterior epidermal area of the left lateral cerebral hemisphere (dorsolateral prefrontal cortex) and the cathode is attached to the upper side of the right eye. The intensity and conduction time, which are stimulus parameters, are applied at 1 mA for 30 minutes when applying tDCS.^[19,20]^

The CCRT will be conducted by an occupational therapist from the Department of Physical and Rehabilitation Medicine at Chonnam National University Hospital. EA will be conducted by a doctor from the Dongshin Traditional Korean Medicine University Hospital, who has more than 2 years of clinical experience in Korean medicine. tDCS will be conducted by a doctor from the Department of Physical and Rehabilitation Medicine at Chonnam National University Hospital.

All participants will receive treatment once a day for 3 days a week (excluding Saturdays and Sundays) over 8 weeks, giving a total of 24 scheduled therapy sessions at Chonnam National University Hospital.

During the period, routine existing medications for hypertension, diabetes, hyperlipidemia, and other medications do not affect cognitive function will be continued. Other treatments that can affect cognitive function including other rehabilitation programs will not be permitted during this period. All medical devices, including the acupuncture needles, EA stimulator, tDCS, and RehaCom software, will be inspected by the investigators who will record the results of check-ups in the management register.

### 2.10 Outcome measurements

All outcomes, including the primary and secondary outcomes, will be evaluated before the intervention, at the end of the intervention, 8 weeks after the first intervention, and 4 weeks after completion of the intervention. We will exclude participants who completed less than 20 therapy sessions out of the 24 scheduled.

### 2.11 Primary outcome

For evaluation of the efficacy of CCRT alone and EA or tDCS with CCRT on cognitive impairment in patients with cerebrovascular disease, we will check the changes in the LOTCA scale score as the primary outcome for cognitive function. Cognitive function in patients with cerebrovascular disease can be evaluated in detail using the LOTCA. The LOTCA is composed of 6 subscales that are subdivided into 26 items. The 6 subscales are as follows:

1. orientation (2 items);
2. visual perception (4 items);
3. spatial perception (3 items);
4. motor praxis (3 items);
5. visuo-motor organization (7 items); and
6. thinking operations (7 items).

The subscale items are scored using a 4- or 4-point scale and total scores range from 27 to 123. A higher score reflects better cognitive function as assessed using the LOTCA.^[21]^

### 2.12 Secondary outcomes

In this protocol, changes in K-MBI and EQ-5D-3L will be used as secondary outcomes. K-MBI is a modified assessment tool to evaluate the performance in activities of daily living in a Korean population that uses 10 items. The K-MBI items include personal hygiene, bathing, eating, toilet use, ability to climb stairs, ability to dress, bowel/bladder function, walking function, ability to get into and out of a chair/bed, and mobility. The score ranges from 0 to 100 and a higher score reflects better performance.^[22]^

EQ-5D-3L is a self-completed instrument for the assessment of health-related quality of life. Using the EQ-5D-3L, we can measure participants’ health-related quality of life and wellbeing using 5 dimensions: mobility, self-care, usual activities, pain, and anxiety/depression. Each dimension has 3 possible response options corresponding to no problems, some problems, or severe problems. Participants rate their overall health state on a 0 to 100 hash-marked, Vertical Visual Analogue Scale, with a higher score representing a better health state.^[23]^

### 2.13 Study termination

Participants will be excluded from this study if adverse events (AEs) occur or the investigator believes that a procedure may cause AEs during the study.

### 2.14 Incidence of AEs

During the study, all unexpected and unintended signs, symptoms, or diseases refer to AEs. During this period, all AEs reported by participants or the investigator will be recorded in detail, such as when the AEs occur, duration of symptoms, degree of severity (mild, moderate, or severe),^[24]^ any measures related to the AEs, time of AEs disappearance, and any potentially causal relationship between the treatment and AEs. All AEs will be reported to the principal investigator and Institutional Review Board and will be documented.

The possible AEs of EA include nausea, vomiting, skin irritation, bleeding, local hematoma, pallor, sweating or dizziness, fainting, headache, and infection, and AEs of tDCS may be pallor, pain, anxiety, sweating or dizziness, fainting, headache, and itching.

### 2.15 Sample size estimation

Like the other pilot study, we calculated that 15 participants are required in each group, giving a total of 45 participants, in this clinical trial. A 25% of dropout rate was taken into consideration for a sample size of 12 per group.^[25]^ The CRC will manage the treatment schedule of subjects to minimize the dropout rate.

### 2.16 Data management

The participants in this study will be required to voluntarily sign the consent form before beginning this protocol; the data will then be collected. All data from this study will be recorded and documented or recorded in Excel files by the CRC and all collected data will only be used for research purposes. Personal data including name and resident registration number will not be recorded or used. For data security, all data will be secured in a double locked cabinet until the end of this study and will only be accessible by investigators. Investigators who monitor the data include the principal investigator, resident in charge of EA, occupational therapist in charge of CCRT, doctor in charge of tDCS, and the CRC.

### 2.17 Statistical analysis

The statistical analysis will be conducted by a statistician who is not involved in this study and SPSS version 21 software (IBM Corp, Armonk, NY) will be used. We will analyze the general characteristics and outcome data at baseline, at the end of the intervention, and 4 weeks after intervention completion. Continuous variables will be presented as means and standard deviations or means and 95% confidence intervals. Independent *t*-test or Wilcoxon rank sum test will be used for continuous data and chi-squared test or Fisher exact test will be used for categorical data. The primary and secondary outcomes of the 3 groups will be measured using the changes in score at the end of the intervention and 4 weeks after the end of the intervention relative to the initially evaluated score. Each group will then be analyzed using a paired *t*-test or Wilcoxon signed rank test. A repeated measures analysis of variance, 2 sample *t*-test, Wilcoxon rank sum test can be used for the analysis of the degree of change at each outcome evaluation period. *P*-values <0.05 will be considered statistically significant.

## 3 Discussion

This protocol is designed to explore the synergistic effect of combined EA and CCRT therapy in the treatment and prevention of cognitive impairment caused by cerebrovascular disorders. In this protocol, we follow the Recommendations for Interventional Trials (SPIRIT) and Consolidated Standards of Reporting Trials (CONSORT) 2010 guidelines.^[26]^ For the evaluation of each therapy, the outcome measurements include the LOTCA, K-MBI, and EQ-5D-3L. The LOTCA is one of the most commonly used qualitative tools in the evaluation of cognitive function in cerebrovascular disease. Therefore, we will use changes in the LOTCA scale as the primary outcome, with changes in the K-MBI and EQ-5D-3L scores as secondary outcomes. Through this clinical trial, we expect to find that EA or tDCS with CCRT is safe and effective for patients with VCI.

This protocol has some limitations. First, we cannot perform a double blinded study because the practitioners know which procedure is applied to which participant. Second, we cannot use a multi-center pilot study and large sample size design due to the limited research funds. Third, we cannot apply another procedure other than EA and tDCS due to the small sample size and lack of adequate preliminary studies.

## Acknowledgments

The authors would like to express sincere thanks to our colleagues and staff at Chonnam National University Medical School and Hospital for their support.

## Author contributions

JH Kim and JY Han are responsible for conceiving and designing the trial, planning data analysis, drafting the manuscript, making the final decision to terminate the trial, and approving the final manuscript. HK Park and MK Song will participate in data collection and are in charge of recruitment and treatment of participants. HK Park and MK Song are responsible for planning data analysis and analyzing the data resulting from the trial. All authors have read and approved the final manuscript.
